# Supplementary material for: Revealing Hidden Genes in Botrytis cinerea: New Insights into Genes Involved in the Biosynthesis of Secondary Metabolites
Source: Int J Mol Sci. 2024 May 28;25(11):5900. doi: 10.3390/ijms25115900 (PMC11173184; doi:10.3390/ijms25115900)
Supplement: Supplementary file 1 [file ijms-25-05900-s001.zip › ijms-2980432-supplementary.pdf]

Supplemetary Materials

Revealing Hidden Genes in *Botrytis cinerea*: New insights into genes involved in the biosynthesis of secondary metabolites.

Ivonne Suárez[1,2,3], Isidro G. Collado[2,3]* and Carlos Garrido[1,4]*

[1] Laboratorio de Microbiología, Departamento de Biomedicina, Biotecnología y Salud Pública, , Facultad de Ciencias del Mar y Ambientales, Universidad de Cádiz, 11510, Puerto Real, Spain. (I.R.S.C. Ivonne.suarez@uca.es)
[2] Departamento de Química Orgánica, Facultad de Ciencias, Campus Universitario Río San Pedro s/n, Torre sur, 4ª planta, Universidad de Cádiz, 11510, Puerto Real, Cádiz, Spain.
[3] Instituto de Investigación en Biomoléculas (INBIO), Universidad de Cádiz, 11510 Puerto Real, Cádiz, Spain.
[4] Instituto de Investigación Vitivinícola y Agroalimentaria (IVAGRO), Universidad de Cádiz, 11510 Puerto Real, Cádiz, Spain.

* Correspondence: Isidro G. Collado (isidro.gonzalez@uca.es); Carlos Garrido (carlos.garrido@uca.es)

This study was conducted by searching for each specific gene encoding enzymes related to the secondary metabolism of *B. cinerea* (B05.10), as currently known [1], in the Ensembl Fungi portal by entering the corresponding Gene ID for each gene. Upon completing the search and entering the Gene ID, the

platform displays three tabs at the top of the page (Location, Gene, and Transcript), allowing the user to switch between these different levels. Additionally, it contains a menu on the left side of the page, which is dependent on the top tabs and offers access to a selection of data views specific to each of these levels [2].

By navigating to the Transcript tab of the gene, the left-hand menu displays various transcription-based data. One of these is Protein Information, which contains five subsections (Protein summary, Domains & features, Variants, PDB 3D protein model, and AlphaFold predicted model). Clicking on Domains & features reveals information regarding the domains present in the queried gene, allowing access to each domain and enabling the consultation of additional genes belonging to that domain (Figure S1).

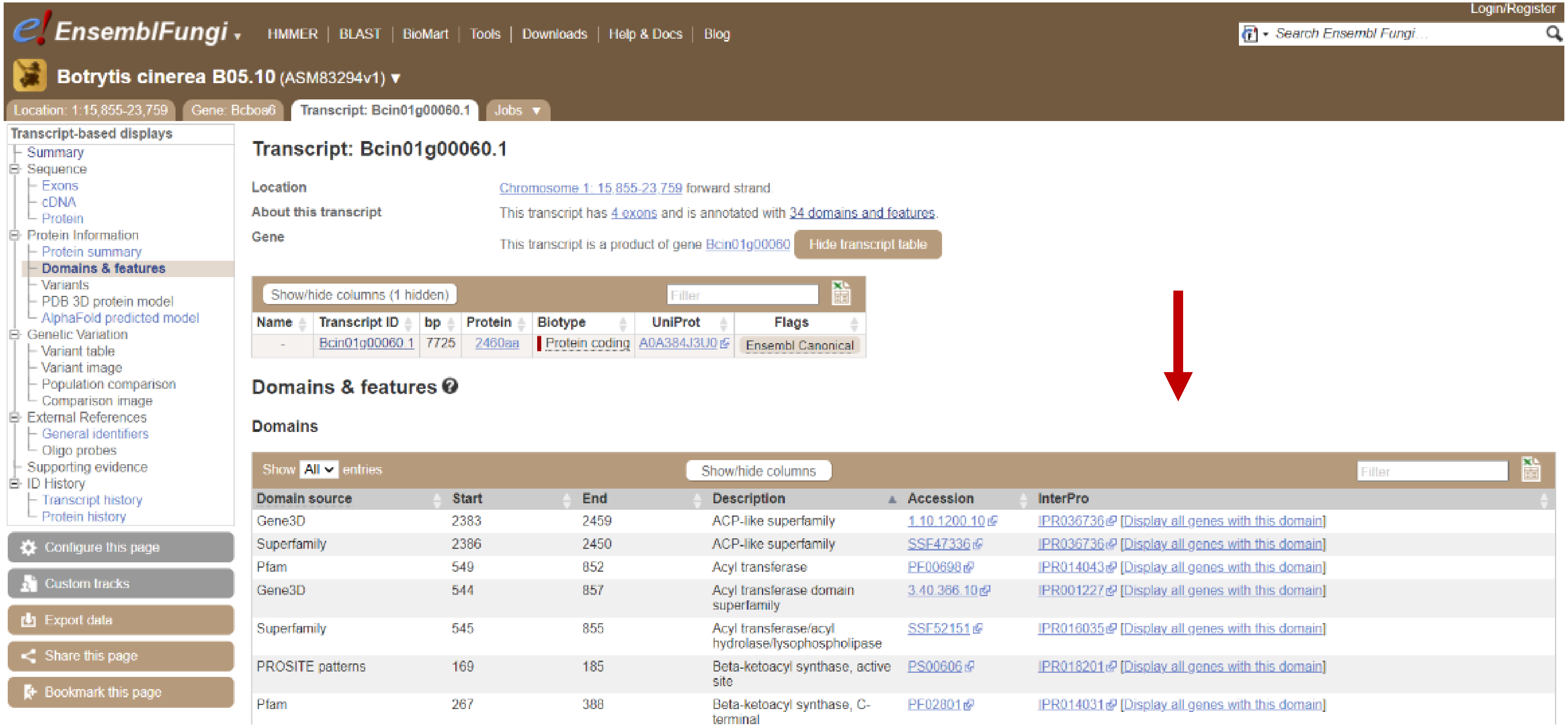

Figure S1. Domains of genes related to the secondary metabolism of *B. cinerea* in the Ensembl Fungi database. (Gene *Bcboa6*, Bcin01g00060)

By clicking on and entering each of these domains, the genes of *B. cinerea* (B05.10) classified within that domain are displayed. For example, clicking on the hyperlink "display all genes with this domain" for the gene *Bcboa*6, for the domain IPR036736, which is the first option shown in Figure S1, opens a new window that displays all genes classified within this domain, as illustrated in Figure S2.

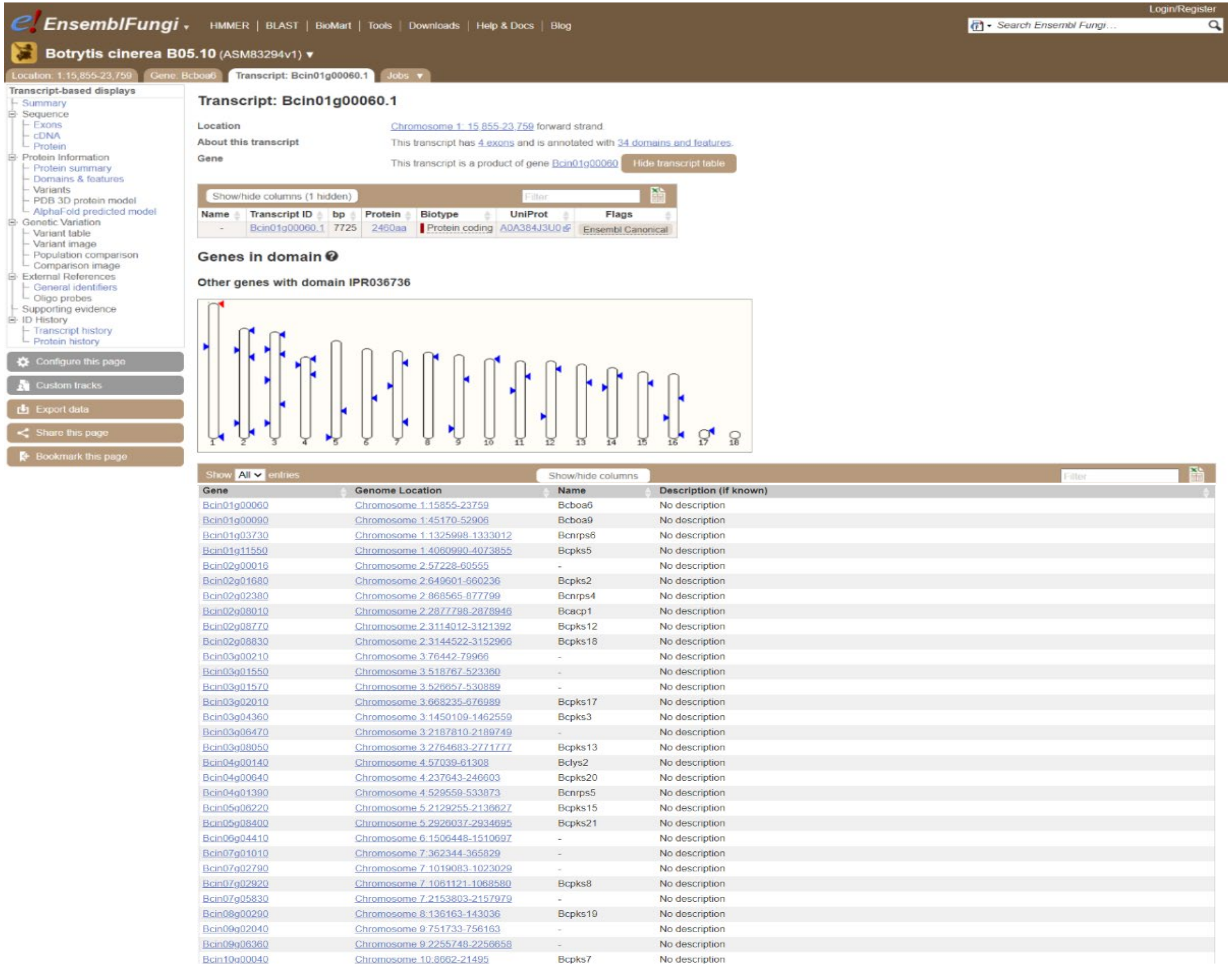

Gene	Genome Location	Name	Description (if known)
Bcin01g00060	Chromosome 1:15855-23759	Bcboa6	No description
Bcin01g00090	Chromosome 1:45170-52906	Bcboa9	No description
Bcin01g03730	Chromosome 1:1325998-1333012	Bcnrps6	No description
Bcin01g11550	Chromosome 1:4060990-4073855	Bcpks5	No description
Bcin02g00016	Chromosome 2:57228-60555	-	No description
Bcin02g01680	Chromosome 2:649601-660236	Bcpks2	No description
Bcin02g02380	Chromosome 2:868565-877799	Bcnrps4	No description
Bcin02g08010	Chromosome 2:2877798-2878946	Bcacp1	No description
Bcin02g08770	Chromosome 2:3114012-3121392	Bcpks12	No description
Bcin02g08830	Chromosome 2:3144522-3152966	Bcpks18	No description
Bcin03g00210	Chromosome 3:76442-79966	-	No description
Bcin03g01550	Chromosome 3:518767-523360	-	No description
Bcin03g01570	Chromosome 3:526657-530889	-	No description
Bcin03g02010	Chromosome 3:668235-676989	Bcpks17	No description
Bcin03g04360	Chromosome 3:1450109-1462559	Bcpks3	No description
Bcin03g06470	Chromosome 3:2187810-2189749	-	No description
Bcin03g08050	Chromosome 3:2764683-2771777	Bcpks13	No description
Bcin04g00140	Chromosome 4:57039-61308	Bclys2	No description
Bcin04g00640	Chromosome 4:237643-246603	Bcpks20	No description
Bcin04g01390	Chromosome 4:529559-533873	Bcnrps5	No description
Bcin05g06220	Chromosome 5:2129255-2136627	Bcpks15	No description
Bcin05g08400	Chromosome 5:2926037-2934695	Bcpks21	No description
Bcin06g04410	Chromosome 6:1506448-1510697	-	No description
Bcin07g01010	Chromosome 7:362344-365829	-	No description
Bcin07g02790	Chromosome 7:1019083-1023029	-	No description
Bcin07g02920	Chromosome 7:1061121-1068580	Bcpks8	No description
Bcin07g05830	Chromosome 7:2153803-2157979	-	No description
Bcin08g00290	Chromosome 8:136163-143036	Bcpks19	No description
Bcin09g02040	Chromosome 9:751733-756163	-	No description
Bcin09g06360	Chromosome 9:2255748-2256658	-	No description
Bcin10g00040	Chromosome 10:8662-21495	Bcpks7	No description

Figure S2. List of genes of the IPR036736 domain (description: ACP-like superfamily; Domain source: Gene 3D) for the *Bcboa6* gene of *B. cinerea* on the Ensembl Fungi database.

Thus, by examining each of the known genes related to secondary metabolism that have been published and annotated to date and observing the domains to which they belong, an initial classification of all domains pertaining to each group of key enzymes was performed and organized into tables, namely, PKS (Table 1), NRPS (Table 3), STC (Table 5). For DTC and DMAT, since they contain only one and two domains respectively, they are listed in the corresponding section.

Additionally, by accessing the Ensembl Fungi platform, the biological processes, molecular functions, and paralogous genes for each of the genes were consulted. Furthermore, to complete the information for each of these genes, additional searches were conducted in other databases such as FungiDB and NCBI (Figure S3). In the former, parameters related to secondary metabolism, such as prediction function and the metabolic pathway involved, were consulted, and in the latter, conserved domains of mRNA and the proteins they encode were examined.

Through this strategy, tables were created listing both known and unknown genes for each of the domains consulted, including information found in the FungiDB and NCBI databases, obtaining a total of 5 tables: Table 2. Genes located in the domains that code for possible PKSs in *B. cinerea*, Table 4. Genes located in the domains that code for possible NRPSs in *B. cinerea*, Table 6. Genes located in the domains that code for possible STCs in *B. cinerea*, Table 7. Genes located in the domains that code for possible DTCs in B. cinerea and Table 8. Genes located in the domains that code for possible DMATS in *B. cinerea*.

The genome contained in the Ensembl Fungi database for the *B. cinerea* B05.10 strain corresponds to Taxonomy ID 332648, Assembly ASM83294v1 (January 2015), INSDC Assembly GCA_000143535.4, February 2015, accession GCA_000143535.4. Data source: Wageningen University, Syngenta.

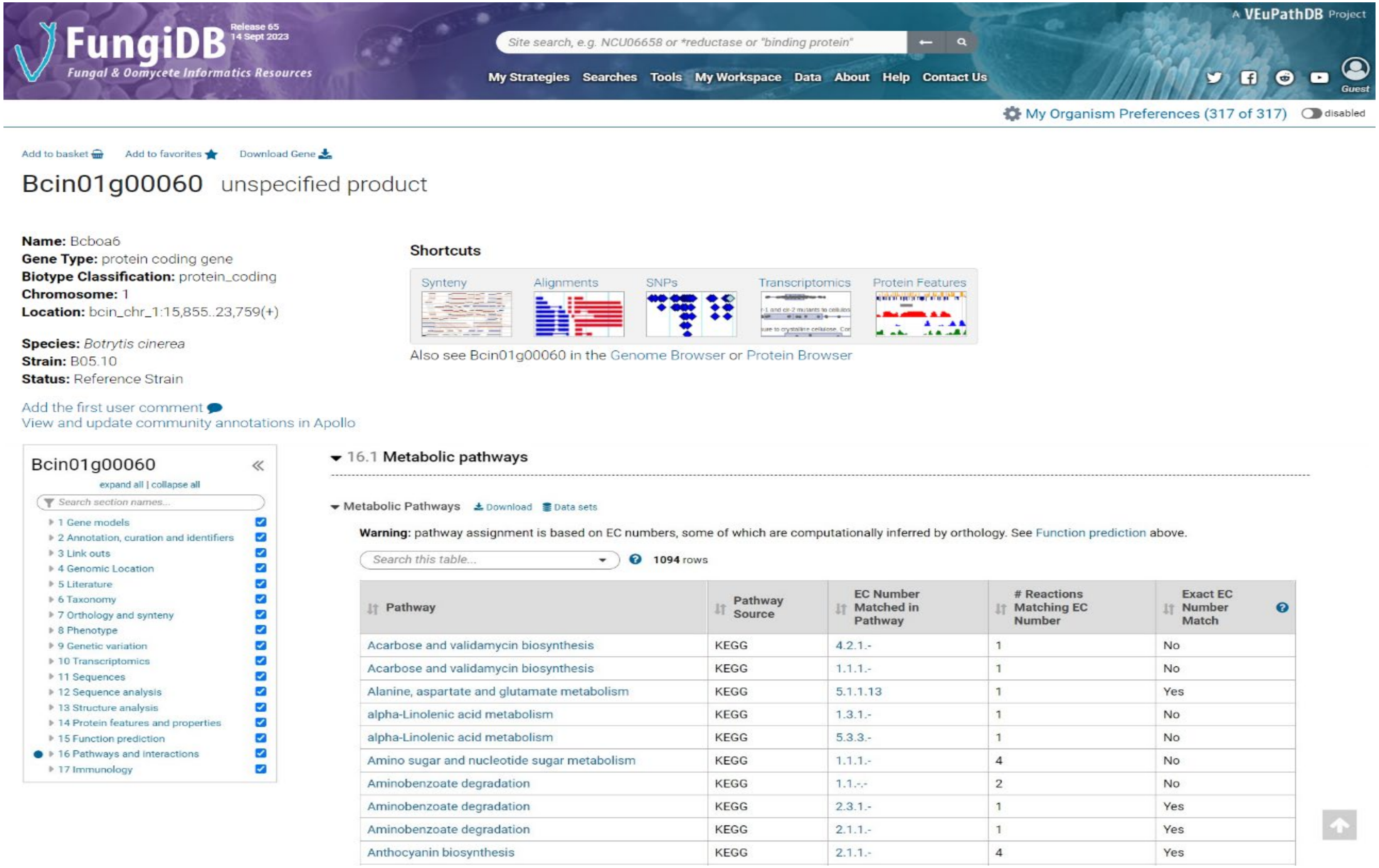

Figure S3. FungiDB database (metabolic pathways Gene *Bcboa6*, Bcin01g00060).

1. da Silva Ripardo-Filho, H.; Coca Ruíz, V.; Suárez, I.; Moraga, J.; Aleu, J.; Collado, I.G. From Genes to Molecules, Secondary Metabolism in *Botrytis cinerea*: New Insights into Anamorphic and Teleomorphic Stages. Plants 2023, 12, 1–31, doi:10.3390/plants12030553.

2. Kersey, P.J.; Lawson, D.; Birney, E.; Derwent, P.S.; Haimel, M.; Herrero, J.; Keenan, S.; Kinsella, R.J.; Kulesha, E.; Maheswari, U.; et al. Ensembl Genomes: Extending Ensembl across the taxonomic space. Nucleic Acids Res. 2010, 38, 563–569, doi:10.1093/nar/gkp871.
